# N-Acetylcysteine Reduces Tissue Injury Induced by Oxygen–Glucose Deprivation in an Organotypic Culture of Mouse Cerebral Cortex Slices

**DOI:** 10.3390/children13030379

**Published:** 2026-03-07

**Authors:** Claudia Villani, Angelo Di Clemente, Roberto William Invernizzi, Rossano Rezzonico

**Affiliations:** 1 Laboratory of Neurochemistry and Behavior, Department of Neuroscience, Istituto di Ricerche Farmacologiche Mario Negri IRCCS, Via Mario Negri 2, 20156 Milan, Italy; claudia.villani@marionegri.it (C.V.); angelo.diclemente@marionegri.it (A.D.C.); 2 Department of Clinical Epidemiology, Istituto di Ricerche Farmacologiche “Mario Negri” IRCCS, Via Mario Negri 2, 20156 Milan, Italy; rossano.rezzonico@marionegri.it

**Keywords:** neonatal hypoxia, mouse, organotypic brain culture, brain injury, influence of sex, glutathione

## Abstract

**Highlights:**

**What are the main findings?**

• In an in vitro model of hypoxic–ischemic cell death, N-acetylcysteine exerts a direct, concentration-dependent cytoprotective effect on cerebral cortex slices whether administered before or after oxygen–glucose deprivation.
• N-acetylcysteine is more potent in reducing cellular damage in cerebral cortex slices derived from female mice than in those from male mice. This effect is associated with an increase in total glutathione levels in the tissue.

**What are the implications of the main findings?**

• It is anticipated that systemic doses of N-acetylcysteine capable of achieving brain drug concentrations similar to those that are cytoprotective in vitro may be effective in in vivo models of hypoxia. This marks a further step toward the potential application of N-acetylcysteine to reduce hypoxia-induced brain damage in newborns.

**Abstract:**

Background/Objectives: Hypoxic–ischemic encephalopathy is the leading cause of infant mortality and disability. Hypothermic therapy is effective in hypoxic–ischemic encephalopathy, albeit in a limited number of cases. Hypothermia requires advanced technologies and significant financial resources, which are difficult to sustain in low-income countries, with devastating consequences. Valid alternatives to hypothermia therapy are therefore needed. Methods: In vitro organotypic cultures of mouse cerebral cortex slices were used to demonstrate the direct protective effect of N-acetylcysteine (NAC) against brain tissue damage induced by oxygen–glucose deprivation (OGD), and to identify the concentrations and time window that maximize the drug’s effectiveness. NAC’s effectiveness was measured by the incorporation of propidium iodide (PI), a marker of cell membrane integrity. Results: Adding 0.1 and 1 mM NAC to the incubation medium before OGD strongly reduced OGD-induced PI incorporation, by 80% (*p* < 0.0002) and 89% (*p* < 0.0001), respectively. Administration of 1 mM NAC 1 h after OGD maintained a high degree of protection against OGD-induced damage (80% reduction in PI incorporation; *p* < 0.0001), while at 0.1 mM, the efficacy of NAC dropped to 44% (*p* < 0.005). Administration of NAC 4 h after OGD reduced PI incorporation to 52% (*p* < 0.005) at 1 mM, while at 0.1 mM, the effect was not significant (17%; *p* > 0.05). Exposure of slices to 0.1 and 1 mM NAC reduced PI incorporation in female cerebral cortex slices (*p* < 0.006), while only the higher concentration was effective in male slices (*p* < 0.05). Exposure to 0.1 mM NAC increased tissue levels of total glutathione (*p* = 0.0185), while no significant effect was observed with 1 mM NAC. Conclusions: This work highlights the direct effect of NAC in protecting cerebral cortex cells from OGD-induced damage and identifies the concentrations and time window that maximize the drug’s effect. The results underscore the need for further studies to verify the in vivo efficacy of NAC at concentrations found to be active in vitro, and for clinical trials to evaluate whether NAC can reduce hypoxia-induced brain damage in newborns.

## 1. Introduction

Hypoxic–ischemic encephalopathy (HIE) is one of the leading causes of death and neurologic disability in neonates [1]. In high-income countries, where high-tech neonatal intensive care units are available, hypothermia therapy has been shown to be effective in reducing mortality, brain injury, and disability in term infants with moderate-to-severe HIE [2,3]. The few studies that have examined low- and middle-income countries [4] have indicated that the composite outcome of death or disability at 18–22 months post-partum is not reduced by hypothermia therapy, and found that mortality is significantly increased [5,6]. Moreover, no benefits or potential adverse outcomes were observed in pre-term neonates, and it remained unclear whether hypothermia benefits neonates with mild HIE and HIE associated with infections [7,8]. For these reasons, effective therapies that can be applied where hypothermia is not available, or is ineffective or contraindicated, remain an unmet need. This spurs research into treatments with drugs known to be effective, easy to administer and store, and low-cost.

In the years prior to the use of hypothermia, numerous neonatal pharmacological treatments were studied and proposed to be administered to the newborn or, where possible, preventively to the mother [9,10]. However, clinical trials on such drugs, some of which have shown some efficacy [11], have virtually ceased since hypothermia therapy became the standard treatment.

In view of the role of oxidative stress, inflammation, and excitotoxicity in brain damage caused by hypoxia–ischemia, it has been proposed that pharmacological suppression of these mechanisms may protect the fetal and neonatal brain from hypoxic–ischemic insult [9,12,13]. N-acetylcysteine (NAC), a well-known antioxidant and anti-inflammatory agent [13], has been used for several decades as a mucolytic and in the treatment of acetaminophen intoxication. NAC crosses the blood–brain and placental barriers [14,15,16] and has the ability to stimulate glutathione production [17]; reduce free radicals, oxidative stress, and inflammatory cytokine expression; and modulate apoptosis [16,18]. Moreover, its intake in pregnancy and by immature infants is considered safe [19,20]. These properties suggest that NAC could be employed to reduce brain injury in neonatal HIE. Maternal administration of NAC prevents hypoxia–ischemia-induced brain damage in neonatal rats [21]. In addition, combination therapy with NAC and hypothermia, and with NAC, hypothermia and vitamin D, attenuates infarct volume and improves functional outcome in HIE neonates and newborn rats undergoing neonatal hypoxia–ischemia [22,23,24,25]. NAC administration to pregnant female rats also shows neuroprotective effects in experimental models of chorioamnionitis, an acute inflammation of the amniotic and chorionic membranes caused by bacterial infection that may lead to white matter damage and neurological sequelae [26,27]. In line with pre-clinical evidence, clinical studies in pregnant women with established chorioamnionitis show that maternal NAC reaches and protects the fetus from damage caused by inflammation [16,28].

Although previous studies have shown NAC to have a consistent protective effect in various experimental models of hypoxia-induced brain damage [21,22,23,26], the optimal timing and dosage of NAC, crucial to the success of the therapy, have not been assessed. The in vitro approach used in this study allows controlling the concentration of NAC that is in direct contact with brain tissue, and, to our knowledge, has never been used in previous studies on the effects of NAC in hypoxia models. Thus, the main aim of the present study was to identify the concentrations of NAC that reduce brain injury induced by oxygen–glucose deprivation (OGD) and the time window that maximizes NAC efficacy.

## 2. Materials and Methods

### 2.1. Experimental Subjects and Study Design

Breeding pairs, consisting of one male and one female C57BL/6J mouse, were purchased from Charles River Laboratories (Calco, Italy). Mice were maintained in a specific pathogen-free animal facility under a 12 h light/dark cycle (light on at 7:00 a.m.) at 21 ± 2 °C and 55 ± 5% relative humidity, with food (Teklad Global, 2018S; Envigo, Udine, Italy) and filtered tap water freely available. Nesting material (wood wool) was provided in breeding cages.

**Figure 1:**
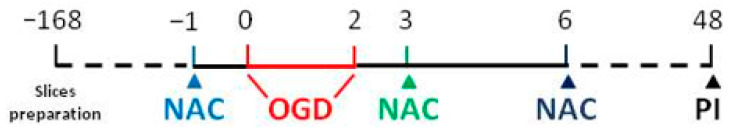
Schematic representation of the experimental protocol. The numbers in the top row indicate the time in hours of the events listed in the bottom row. NAC was administered 1 h before or 1 or 4 h after OGD, as highlighted by the different colors. OGD, oxygen–glucose deprivation; NAC, n-acetylcysteine; PI, propidium iodide incorporation.

The experimental groups were the following: control (CTR; no treatment), OGD, NAC 0.1 mM + OGD, and NAC 1 mM + OGD. NAC was added to the culture medium 1 h before or 1 or 4 h after OGD (Figure 1).

A total of 77 pups were used in 8 different experiments. On average, 2–4 slices of cerebral cortex were obtained per mouse. Less than 25% of the slices were discarded, either because they were damaged, non-viable or included extra-cortical brain regions. Each experiment was repeated twice, with the exception of the experiment examining the effect of NAC administered 4 h after OGD, which was done only once.

### 2.2. Preparation of Cerebral Cortex Slice Cultures

Mouse pups aged 2–4 days (P2–P4) were sacrificed, and the brain was removed from the skull under sterile conditions. The tissue was embedded in a 3% agar solution at 37 °C and cooled down to room temperature. The ventral part of the forebrain was discarded. The tissue was fixed onto the stage of the vibratome (Leica, VT 1000S; Leica Biosystems, Buccinasco, Italy) with cyanoacrylic glue and placed in an ice-cold artificial cerebral spinal fluid (aCSF) solution (87 mM NaCl, 25 mM NaHCO_3_, 1.25 mM NaH_2_PO_4_, 7 mM MgCl_2_, 0.5 mM CaCl_2_, 2.5 mM KCl, 25 mM D-glucose, 75 mM sucrose, 50 U/mL penicillin, and 50 μg/mL streptomycin, equilibrated with 95% O_2_ and 5% CO_2_, pH = 7.4). Starting from the front pole, 4 coronal sections (200 μm thick) were cut and transferred into Petri dishes filled with ice-cold aCSF. Cortical slices were placed on membranes of Millicell Culture insert with 0.4 μm pore size (Merck-Millipore, Darmstadt, Germany), with two to four slices per insert, and placed in 6-well plates. Although no actual randomization was planned, the cortical slices of each mouse were distributed evenly among the four experimental conditions, thereby minimizing allocation bias. Each well was filled with 1 mL culture medium containing 25% MEM-Glutamax (Thermo Fisher Scientific, Monza, Italy), 25% basal medium eagle (Thermo Fisher Scientific, Monza, Italy), 25% horse serum (Euroclone, Pero, Italy), 0.6% glucose, 100 U/mL penicillin, and 100 μg/mL streptomycin (Euroclone, Pero, Italy) with a pH of 7.2. All the cultures were maintained at 37 °C in O_2_/CO_2_ (95/5). The incubation medium was replaced with freshly prepared NB/B27 medium (Thermo Fisher Scientific, Monza, Italy) every two days.

### 2.3. Hypoxic Chamber

After one week in culture, slices were washed twice with PBS and transferred into a temperature-controlled (37° ± 1 °C) hypoxic chamber (InvivO_2_ 400, Baker Ruskinn, Sanford, ME, USA) at [O_2_] = 0.1%, [CO_2_] = 5%, and [N_2_] = 95%, and the PBS was replaced with deoxygenated glucose and pyruvate-free medium at 37 °C. After 2 h OGD, cerebral cortex slices were returned to a normoxic incubator, and the medium was replaced with NB/B27. Control slices were maintained in a normoxic incubator with NB/B27 medium.

### 2.4. Assessment of Cell Viability

Cell viability was assessed by the incorporation of propidium iodide (PI) into cerebral cortex slices [29]. Slice images were acquired using an IX-51 microscope (Olympus, Segrate Italy). A 4× objective coupled to a PH2 phase annulus was used for morphological visualization and to measure the slice surface. PI fluorescence was detected using a green excitation filter (532–561 nm range), consistent with its spectral properties. Exposure time was fixed at 50 ms, and all acquisition parameters (illumination intensity, detector gain, and camera settings) were predefined, kept identical across groups and independent replicates, and applied under non-saturating conditions. No post hoc adjustments of acquisition parameters were performed.

Quantitative analysis was performed using ImageJ 1.51j8 (NIH). Images were spatially calibrated based on microscope settings (310 pixels = 1 mm) and converted to 8-bit. Integrated density (IntDen) was measured with the “limit to threshold” option enabled. Background levels were assessed by selecting multiple regions devoid of PI signal, and threshold values were defined using the “Dark Background” setting. This workflow, including calibration, thresholding, and measurement, was applied consistently across all images without group-specific adjustments, ensuring reproducibility and minimizing observer bias.

The fluorescence signal emitted by PI was normalized for the slice area (in mm^2^), and PI incorporation was expressed as a percentage of the OGD-group values.

PI incorporation was measured separately in each slice of the left and right hemisphere. The average value of PI incorporation in both hemispheres was used for statistical analysis. The percentage of protection afforded against OGD-induced PI incorporation was calculated according to the following equation: OGD−(NAC + OGD)/OGD-CTR.

### 2.5. Determination of Tissue Levels of Glutathione

Total glutathione levels were measured using the Glutathione Assay Kit CS0260 (Merck Life Science S.r.l., Milano, Italy). Cerebral cortex slices were placed in 1.5 mL Eppendorf tubes with 100 µL 5% sulfosalicylic acid and tissue homogenized by sonication while kept chilled on minced ice. Samples were centrifuged at 10,000× *g* for 10 min at 4 °C. A total of 10 μL supernatant was transferred to a 96-well plate, and samples were processed according to the manufacturer’s instructions. Aliquots of the homogenate were used for the determination of proteins using the Pierce BCA assay kit (Thermo Fisher Scientific, Monza, Italy).

### 2.6. Outcome and Data Analysis

The primary outcome was cell damage, defined as the incorporation of PI into cellular DNA in sections of cerebral cortex. Differences between genders in the protective effect of NAC and tissue levels of glutathione were also evaluated.

Using GraphPad Prism, version 10.1.2 (GraphPad Software, Boston, MA, USA), the results were analyzed by one-way ANOVA followed by Dunnett’s test for post hoc comparisons between OGD and the other experimental groups. The same tests were used to analyze glutathione levels in various experimental groups.

## 3. Results

### 3.1. Effect of NAC on PI Incorporation into Cerebral Cortex Slices

By itself, NAC had no effect on PI incorporation (Table 1), except at 10 mM, which significantly increased it (F3,43 = 4.754; *p* = 0.006; one-way ANOVA). For this reason, 10 mM NAC was excluded from subsequent experiments.

### 3.2. Effect of NAC on OGD-Induced Increase in PI Incorporation into Cerebral Cortex Slices

**Figure 2:**
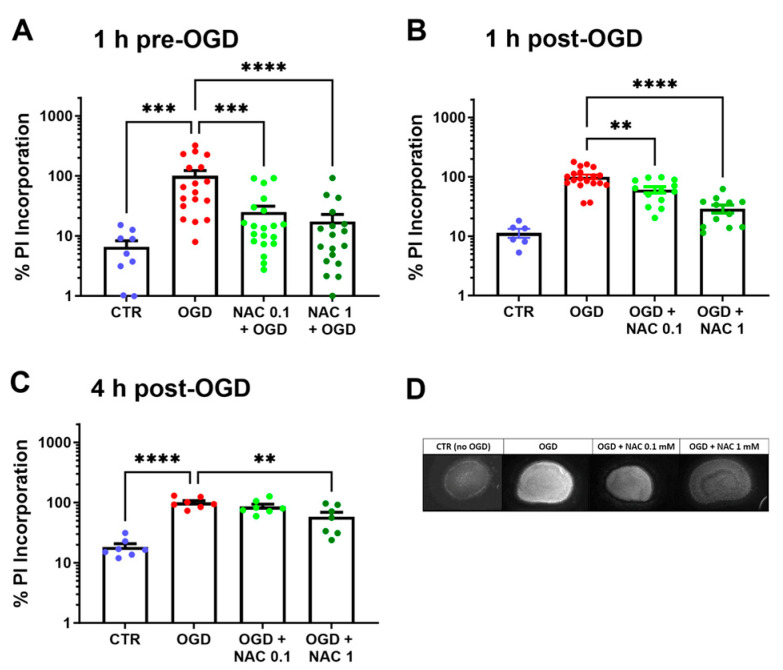
Effect of NAC on PI incorporation in cerebral cortex slices: panel (**A**), effect of NAC 1 h before OGD; panel (**B**), effect of NAC 1 h after OGD; and panel (**C**), effect of NAC 4 h after OGD. Representative pictures showing PI incorporation into cerebral cortex slices exposed to OGD, with and without NAC pre-treatment and controls (CTR) are shown in panel (**D**). Data are the mean ± SEM and are expressed as a percentage of the OGD values. ** *p* < 0.005, *** *p* < 0.0002, **** *p* < 0.0001 vs. OGD (Dunnett’s test).

Figure 2A shows the effect of NAC added to culture medium 1 h before OGD on PI incorporation in cerebral cortex slices. OGD significantly increased PI incorporation, and this effect was reduced by 80% and 89%, respectively, by 0.1 and 1 mM NAC. One-way ANOVA showed a highly significant F value (F3,61 = 9.885; *p* < 0.0001). Figure 2B shows that protection against hypoxic injury remains robust even if NAC is administered 1 h after OGD: a 44% and 80% reversal of PI incorporation were achieved at 0.1 and 1 mM, respectively (F3,46 = 22.50; *p* < 0.0001). Figure 2C shows that the results for the administration of NAC 4 h after OGD were significant (F3,24 = 19.36; *p* < 0.0001), albeit with a partial reduction in PI incorporation (17% and 52% with 0.1 and 1 mM, respectively). A representative picture of PI incorporation into cerebral cortex slices is shown in Figure 2D.

### 3.3. The Effect of NAC on Tissue Damage Induced by OGD Is Influenced by Sex

**Figure 3:**
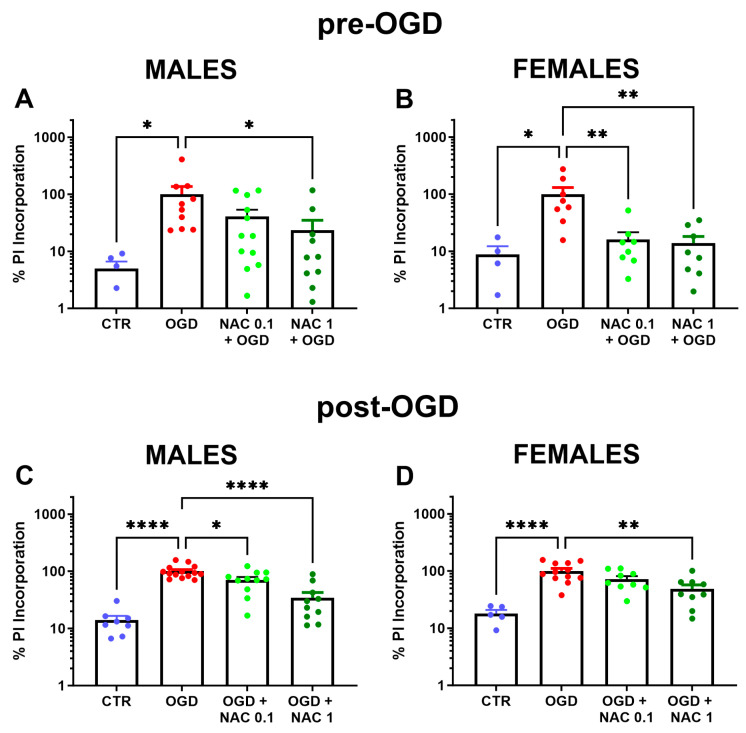
Sex influence on the effects of NAC on OGD-induced PI incorporation. Panels (**A**,**B**) show the effect of NAC given 1 h before OGD on PI incorporation in cerebral cortex slices in males and females, respectively. Panels (**C**,**D**) show the effect of NAC given after OGD on PI incorporation in males and females, respectively. Data are the mean ± SEM and are expressed as a percentage of the OGD values. * *p* < 0.05, ** *p*< 0.006, **** *p* < 0.0001 vs. OGD (Dunnett’s test).

Figure 3 shows the effect of NAC added to the culture medium before and after OGD on PI incorporation into cerebral cortex slices in male and female mice. The addition of 0.1 and 1 mM NAC to the culture medium prior to OGD reduced PI incorporation in cerebral cortex slices by 92% and 95%, respectively, in female mice (F3,24 = 6.037; *p* = 0.0033). In male mice slices, 1 mM NAC was required to reduce OGD-induced PI incorporation by 80%, while at 0.1 mM, NAC reduced PI incorporation by 62% (F3,33 = 3.009; *p* = 0.044). Because the size of some experimental groups was too small (n = 2–3), the influence of sex on the effect of NAC administration after OGD was analyzed after pooling the data obtained at 1 and 4 h. The results showed that NAC treatment after OGD reduced cellular damage to a similar extent in cerebral cortex slices derived from males (34 and 71% after 0.1 and 1 mM NAC, respectively) and females (34 and 63% after 0.1 and 1 mM NAC, respectively). ANOVA showed significant effects in males (F3,39 = 25.14; *p* < 0.0001) and females (F3,31 = 10.70; *p* < 0.0001).

### 3.4. Effect of NAC on Total Glutathione Levels in Cerebral Cortex Slices

The effect of 0.1 and 1 mM NAC added to the incubation medium 1 h after OGD on total glutathione levels in cerebral cortex slices is shown in Table 2.

Compared to OGD, NAC increased total glutathione levels by 84% and 52% at 0.1 and 1 mM, respectively. Overall, ANOVA showed a significant effect (F4,39 = 4.039; *p* = 0.0258). Post hoc comparisons showed a significant increase in glutathione levels in the NAC 0.1 mM + OGD group compared to CTR and a nearly significant effect when the former was compared to OGD (*p* = 0.0543). NAC 1 mM + OGD had no significant effects on glutathione levels. Glutathione levels in CTR and OGD were not significantly different.

## 4. Discussion

This study shows that NAC protects brain tissue from OGD-induced cell damage in a concentration-dependent manner. NAC was effective not only when administered before OGD, but also when given after the insult. NAC was more effective in reducing hypoxic damage in slices of cerebral cortex derived from female mice.

At 0.1 mM, NAC was sufficient to completely prevent OGD-induced hypoxic damage. However, the effect was short-lived, and 0.1 mM NAC was only partially effective or completely ineffective when administered after OGD. At 1 mM, however, NAC provided cytoprotection even when administered after OGD. This pattern probably reflects the rapid clearance of NAC from the culture medium [30] and suggests that increasing NAC concentrations prolongs the protective effect of NAC, thereby compensating for NAC’s short duration of action. Thus, in order to maintain protective concentrations of NAC over time, the administration protocol must be carefully optimized in future in vivo experimental studies and clinical trials, preferably, for example, by using continuous infusion rather than spaced doses.

The NAC concentrations that reduce OGD-induced cell damage in our study are similar to those of NAC-amide, which protects rat hippocampus slices from oxidative damage caused by kainic acid [31], and are close to the plasma concentrations found in hypoxic and premature newborns receiving NAC intravenously or via placental transfer from mothers treated with NAC following a diagnosis of chorioamnionitis [16]. However, it is not known whether similar concentrations of NAC can reach the brain after systemic administration. In patients with Parkinson’s disease, oral intake of NAC led to drug concentrations in the cerebrospinal fluid of about 10 μM [15]. Higher brain concentrations could probably be achieved with intravenous administration, which leads to circulating levels of NAC 10–20 times higher than achieved through oral administration [32]. It has been estimated that about 0.4% of an NAC dose injected into the mouse jugular vein enters the brain [14]. Therefore, it is conceivable that high doses of NAC (100–150 mg/kg), which have been safely administered intravenously to adults and, via placental transfer, to newborns [28,33], and NAC doses of 300 mg/kg, which reduced hypoxia-induced brain damage in neonatal rats when given to dams or their pups, could achieve brain NAC concentrations similar to those used in vitro in this study. The fact that repeated intravenous infusions of NAC in neonates result in NAC concentrations in the millimolar range in the cerebrospinal fluid confirms this prediction [25].

We did not initially plan to evaluate how gender differences affected the ability of NAC to reduce OGD-induced tissue damage. However, post hoc analysis showed that the greater efficacy of NAC in female mouse cerebral cortex slices in which 0.1 mM NAC was administered before OGD was sufficient to achieve almost total protection from hypoxic damage, while in cerebral cortex slices derived from male mice, higher concentrations of NAC were required to achieve a similar effect. Surprisingly, NAC administration after OGD had similar protective effects in both sexes, suggesting that females may benefit more from early treatment with NAC than males. The increased sensitivity to NAC of female cerebral cortex slices is consistent with previous in vivo studies in rats on the protective effect of NAC against hypoxic–ischemic damage [24,34]. Similarly, hypothermia improved motor and memory deficits induced by hypoxia–ischemia in female rat neonates, but not in males [35]. In contrast, deletion of the poly ADP-ribose polymerase gene rescued neonatal brain damage induced by hypoxia–ischemia only in male mice [36]. NAC enhanced the ability of hypothermia to reduce brain atrophy induced by hypoxic–ischemic insult in neonatal female rats but not in male rats. Interestingly, the addition of vitamin D improved the efficacy of the hypothermia/NAC combination only in male rats [24]. Overall, these findings highlight the role of sex in the response to hypoxia treatments. The underlying mechanism was not addressed in the present study. However, sex differences in the expression of inflammatory and immunological factors have been involved in the response to hypoxic treatments including hypothermia, NAC, and a combination of NAC and hyperthermia [34]. In addition, sexual dimorphism in glutathione biosynthesis and metabolism may also play a role [37]. Because neonatal hypoxia has a worse prognosis in males [38], it is essential that future studies aimed at identifying new treatments for hypoxia take sex into account as a biological variable in order to avoid bias and incorrect conclusions.

It was found that NAC increased glutathione levels in cerebral cortex slices by over 50%, indicating that NAC boosts glutathione synthesis. The magnitude of the NAC-induced increase in glutathione found in the present study was similar to or greater than that observed in the brain and liver of hypoxic–ischemic neonatal rats treated with NAC [21,22]. It remains to be determined whether the increase in glutathione levels is essentially a pharmacodynamic effect, involves the restoration of redox balance, or is the consequence of improved cell viability.

In the present study, OGD did not lower glutathione levels in cerebral cortex slices. This is consistent with previous findings in hippocampus slices [39]. However, hypoxia–ischemia in human neonates and newborn rats reduced brain glutathione levels [22,40]. The average concentration of glutathione measured in the cerebral cortex slices (224 µM in this study) was lower than that measured in freshly dissected whole brain tissue and brain slices (>1 mM) [17,41,42]. Thus, it is conceivable that the already depleted glutathione levels in brain slices could prevent a further reduction caused by OGD. However, it cannot be ruled out that differences between studies, such as the redox state of glutathione, the brain region, gender, or other factors, may have contributed [43,44].

The in vitro approach is the main strength of this study, as it allowed the direct effects of NAC on OGD-induced cell damage to be detected. Another strength is the evaluation, in the same study, of the time window within which NAC protects brain tissue from hypoxic damage. At the same time, the in vitro model is limited by the lack of blood flow, the blood–brain barrier, and the influence of immune response and systemic inflammation, which could affect the effect of NAC. Furthermore, our study focused on the acute effects of OGD, so it is not known whether the observed cytoprotective effect can be maintained in the long term.

## 5. Conclusions

Although the efficacy of NAC in reducing OGD-induced cell damage in vitro is not directly translatable in terms of clinical relevance, our results provide clear evidence of the direct cytoprotective effect of NAC, the concentrations required to achieve it, and the time window within which the effect is maximal. These findings warrant further studies to document the efficacy and pharmacokinetics of NAC in an in vivo mouse model of hypoxia and point to the need to design clinical trials to evaluate whether NAC can reduce hypoxia-induced brain damage, disability, and mortality in newborns with encephalopathy who do not meet current clinical criteria for hypothermic therapy.

## Figures and Tables

**Table 1:** Effect of 0.1, 1.0 and 10.0 mM NAC on PI incorporation in cerebral cortex slices.

Treatment	Concentration	PI Incorporation
		(% of CTRL)
CTR		100 ± 35 (11)
NAC	0.1 mM	130 ± 30 (10)
NAC	1 mM	126 ± 29 (13)
NAC	10 mM	432 ± 147 ** (13)

The data are the means ± SEM and are expressed as percentage of control (CTR) values. The number of samples per group is shown in parentheses. ** *p* = 0.006 vs. CTR (Dunnett’s test).

**Table 2:** Effect of NAC and OGD on glutathione levels in cerebral cortex slices.

Treatment	NAC	Total Glutathione
	Concentration	(nmoles/mg Protein)
CTR		2.8 ± 0.4
OGD		3.4 ± 0.8
OGD + NAC	0.1 mM	6.3 ± 1.1 *
OGD + NAC	1 mM	5.2 ± 0.6

NAC was added to culture medium 1 h after OGD, and slices were harvested 48 h after OGD. The data are means ± SEM of five samples per group. * *p* = 0.0185 vs. CTR.

## Data Availability

The original data presented in the study are available as Supplementary Material.

## Supplementary Materials

The following supporting information can be downloaded at https://www.mdpi.com/article/10.3390/children13030379/s1, raw data relating to the content of Table 1, Figure 2, Figure 3, and Table 2.

## Abbreviations

The following abbreviations are used in this manuscript:

aCSF	Artificial cerebrospinal fluid
ANOVA	Analysis of variance
CTR	Control
HIE	Hypoxic–ischemic encephalopathy
NAC	N-acetylcysteine
OGD	Oxygen–glucose deprivation
P2–P4	Postnatal days 2–4
PBS	Phosphate buffer saline
PI	Propidium iodide
