# Supplementary material for: N-Acetylcysteine Reduces Tissue Injury Induced by Oxygen–Glucose Deprivation in an Organotypic Culture of Mouse Cerebral Cortex Slices
Source: Children (Basel). 2026 Mar 7;13(3):379. doi: 10.3390/children13030379 (PMC13025480; doi:10.3390/children13030379)
Supplement: Supplementary file 1 [file children-13-00379-s001.zip › children-4153178-supplementary.pdf]

Authors: C. Villani, A. Di Clemente, R.W. Invernizzi, R. Rezzonico
Title: N-acetylcysteine reduces tissue injury induced by hypoxia in mouse cerebral cortex slices
Children special issue:
Hypoxic-Ischemic Brain Injury in Newborn: The present status and Challenges in The Future

**Raw data: Table 1**	**% CTR**				**Absolute fluorescence units**				
Sample #	CTR	NAC 0.1 mM	NAC 1 mM	NAC 10 mM	Sample #	CTR	NAC 0.1 mM	NAC 1 mM	NAC 10 mM
1	119,15	127,07	156,89	341,36	1	1,24	1,32	1,63	3,55
2	130,64	116,27	395,89	174,18	2	1,36	1,21	4,12	1,81
3	67,26	128,38	89,57	206,34	3	0,7	1,34	0,93	2,15
4	80,71	117,89	175,08	254,51	4	0,84	1,23	1,82	2,65
5	58,61	259,63	193,42	190,26	5	0,61	2,70	2,01	1,98
6	143,62	198,90	122,99	49,49	6	1,49	2,07	1,28	1,08
7	25,39	116,94	94,17	178,36	7	0,55	2,55	0,98	3,89
8	9,87	13,58	108,36	783,08	8	0,22	0,30	2,36	17,09
9	36,35	5,04	168,27	531,97	9	0,79	0,11	3,67	11,61
10	11,10	23,04	26,78	1606,91	10	0,24	0,50	0,58	35,07
11	417,29	153,50	32,05		11	9,11	3,35	0,70	
12		46,82	28,80		12		1,02	0,63	
13		386,72	42,62		13		8,44	0,93	

Raw data: Figure 2, panel A	% OGD				Absolute fluorescence intensity				
Sample #	CTR	OGD	NAC 0.1 mM + OGD	NAC 1 mM + OGD	Sample #	CTR	OGD	NAC 0.1 mM + OGD	NAC 1 mM + OGD
1	15,17	31,37	91,86	12,02	1	4,73	2,23	6,54	0,86
2	0,65	80,69	10,75	10,48	2	0,20	5,75	0,77	0,75
3	3,75	321,91	14,76	15,73	3	1,17	22,92	1,05	1,12
4	0,87	256,37	13,50	25,02	4	0,27	18,25	0,96	1,78
5	8,92	137,20	14,61	6,64	5	2,78	9,77	1,04	0,47
6	9,12	53,20	41,79	3,45	6	2,84	3,79	2,98	0,25
7	3,13	42,05	91,16	92,52	7	0,97	2,99	6,49	6,59
8	5,08	65,19	4,49	6,13	8	1,58	4,64	0,32	0,44
9	12,62	74,86	30,05	48,18	9	3,93	5,33	2,14	3,43
10		18,81	76,51	43,20	10		1,34	5,45	3,08
11		18,35	7,28	2,09	11		1,31	2,27	0,65
12		38,83	9,54	3,80	12		12,10	2,97	1,18
13		225,00	8,14	2,14	13		70,12	2,54	0,67
14		7,98	15,60	6,85	14		2,49	4,86	2,13
15		139,32	10,10	1,00	15		43,42	3,15	0,31
16		231,12	7,43	14,65	16		72,03	2,31	4,57
17		17,08	2,74	4,86	17		5,32	0,85	1,51
18		40,67	26,30	13,24	18		12,68	8,20	4,13
19			3,49		19			1,09	
20			16,54		20			5,16	

Raw data: Figure 2, panel B	% OGD				Absolute fluorescence intensity				
Sample #	CTR	OGD	OGD + NAC 0.1 mM	OGD + NAC 1 mM	Sample #	CTR	OGD	OGD + NAC 0.1 mM	OGD + NAC 1 mM
1	14,03	162,25	70,87	43,61	1	0,64	19,86	8,67	5,34
2	13,58	102,37	86,54	38,17	2	0,62	12,53	10,59	4,67
3	18,28	146,30	98,30	14,35	3	0,83	17,91	12,03	1,76
4	8,79	101,06	50,17	62,17	4	0,40	12,37	6,14	7,61
5	5,30	73,19	56,43	37,82	5	0,24	8,96	6,91	4,63
6	8,10	93,60	28,93	33,82	6	0,37	11,46	3,54	4,14
7		78,14	97,43	36,95	7		9,57	11,93	1,68
8		118,94	30,78	19,47	8		14,56	1,40	0,89
9		77,97	20,33	14,23	9		9,54	0,93	0,65
10		72,29	40,78	23,60	10		8,85	1,86	1,08
11		152,10	89,47	11,45	11		18,62	4,08	0,52
12		36,85	64,12	13,79	12		4,51	2,92	0,63
13		84,95	58,61		13		10,40	2,67	
14		79,27			14		3,61		
15		178,28			15		8,12		
16		87,66			16		3,99		
17		105,46			17		4,81		
18		35,94			18		1,64		
19		113,40			19		5,17		

Raw data: Figure 2, panel C		% OGD			Absolute fluorescence intensity				
Sample #	CTR	OGD	OGD + NAC 0.1 mM	OGD + NAC 1 mM	Sample #	CTR	OGD	OGD + NAC 0.1 mM	OGD + NAC 1 mM
1	22,73	72,91	59,20	33,48	1	6,57	21,06	17,10	9,67
2	16,88	92,20	82,35	91,95	2	4,88	26,64	23,79	26,56
3	15,12	84,63	79,65	96,62	3	4,37	24,45	23,01	27,91
4	11,95	124,49	75,11	71,07	4	3,45	35,96	21,70	20,53
5	31,21	101,66	126,63	23,79	5	9,02	29,37	36,58	6,87
6	16,41	129,91	105,19	56,06	6	4,74	37,53	30,39	16,19
7	13,60	94,19	71,94	31,02	7	3,93	27,21	20,78	8,96

Raw data: Figure 3, panel A		% OGD			Absolute fluorescence intensity				
Sample #	CTR	OGD	NAC 0.1 mM + OGD	NAC 1 mM + OGD	Sample #	CTR	OGD	NAC 0.1 mM + OGD	NAC 1 mM + OGD
1	9,16	39,86	116,72	15,27	1	4,73	2,23	6,54	0,86
2	0,40	409,05	18,75	19,99	2	0,20	22,92	1,05	1,12
3	2,27	67,60	18,56	4,39	3	1,17	3,79	1,04	0,25
4	5,51	53,43	53,10	117,57	4	2,84	2,99	2,98	6,59
5	7,62	82,84	115,84	7,79	5	3,93	4,64	6,49	0,44
6		23,90	38,19	54,90	6		1,34	2,14	3,08
7		23,32	97,22	2,29	7		1,31	5,45	1,18
8		135,87	5,76	1,30	8		70,12	2,97	0,67
9		139,57	4,92	4,13	9		72,03	2,54	2,13
10		24,56	9,42	8,00	10		12,68	4,86	4,13
11			1,66		11			0,85	
12			9,99		12			5,16	

Raw data: Figure 3, panel B

% OGD					Absolute fluorescence intensity				
Sample #	CTR	OGD	NAC 0.1 mM + OGD	NAC 1 mM + OGD	Sample #	CTR	OGD	NAC 0.1 mM + OGD	NAC 1 mM + OGD
1	1,71	58,78	7,83	7,64	1	0,27	5,75	0,77	0,75
2	17,56	186,75	9,83	18,23	2	2,78	18,25	0,96	1,78
3	6,15	99,94	3,27	4,84	3	0,97	9,77	0,32	0,47
4	9,99	54,53	14,34	35,09	4	1,58	5,33	2,27	3,43
5		76,43	19,89	4,10	5		12,10	3,15	0,65
6		15,71	14,62	1,98	6		2,49	2,31	0,31
7		274,24	51,77	28,84	7		43,42	8,20	4,57
8		33,62	6,86	9,57	8		5,32	1,09	1,51

Raw data: Figure 3, panel C

% OGD					Absolute fluorescence intensity				
Sample #	CTR	OGD	OGD + NAC 0.1 mM	OGD + NAC 1 mM	Sample #	CTR	OGD	OGD + NAC 0.1 mM	OGD + NAC 1 mM
1	11,21	157,62	68,85	42,36	1	0,62	105,01	8,67	5,34
2	15,08	98,17	95,50	32,85	2	0,83	150,08	12,03	4,14
3	7,26	75,91	94,65	16,07	3	0,40	75,08	11,93	0,89
4	6,68	115,55	16,78	11,75	4	0,37	96,02	0,93	0,65
5	16,37	70,22	33,64	19,47	5	4,88	79,99	1,86	1,08
6	11,59	82,53	73,82	11,38	6	3,45	156,03	4,08	0,63
7	30,26	147,10	48,36	89,15	7	9,02	37,80	2,67	26,56
8	13,19	72,32	79,85	68,91	8	3,93	3,99	23,79	20,53
9		87,01	72,83	23,07	9		4,81	21,70	6,87
10		93,56	122,77	30,08	10		5,17	36,58	8,96
11		89,40	69,75		11		26,64	20,78	
12		120,71			12		35,96		
13		98,56			13		29,37		
14		91,33			14		27,21		

Raw data: Figure 3, panel D	% OGD				Absolute fluorescence intensity				
Sample #	CTR	OGD	OGD + NAC 0.1 mM	OGD + NAC 1 mM	Sample #	CTR	OGD	OGD + NAC 0.1 mM	OGD + NAC 1 mM
1	24,35	105,01	88,77	39,16	1	0,64	12,53	10,59	4,67
2	9,19	150,08	51,46	14,72	2	0,24	17,91	6,14	1,76
3	23,72	75,08	57,89	63,78	3	6,57	8,96	6,91	7,61
4	15,78	96,02	29,67	38,80	4	4,37	11,46	3,54	4,63
5	17,13	79,99	53,44	64,15	5	4,74	9,54	1,40	1,68
6		156,03	111,31	19,88	6		18,62	2,92	0,52
7		37,80	61,79	34,94	7		4,51	17,10	9,67
8		137,61	83,13	100,84	8		3,61	23,01	27,91
9		62,39	109,78	58,50	9		1,64	30,39	16,19
10		76,09			10		21,06		
11		88,33			11		24,45		
12		135,58			12		37,53		
12		135.58							

Raw data: Table 2				
Sample #	CTR	OGD	OGD + NAC 0.1 mM	OGD + NAC 1 mM
		nmoles/mg protein		
1	3,32	4,49	5,98	6,41
2	3,52	4,59	9,49	6,30
3	2,78	2,00	3,23	3,54
4	0,99	1,00	4,51	4,40
5	3,51	5,09	8,35	5,39
